# Non-synonymous sequence variants within the oxygen-dependent degradation domain of the *HIF1A *gene are not associated with pre-eclampsia in the Finnish population

**DOI:** 10.1186/1471-2350-9-96

**Published:** 2008-11-03

**Authors:** Sanna Heino, Milja Kaare, Sture Andersson, Hannele Laivuori

**Affiliations:** 1 Department of Medical Genetics, Haartman Institute, FI-00014 University of Helsinki, Finland; 2 Folkhälsan Institute of Genetics, FI-00014 University of Helsinki, Finland; 3 Hospital for Children and Adolescents, Helsinki University Central Hospital, FI-00029 Finland; 4 Department of Pediatrics, FI-00014 University of Helsinki, Finland; 5 Department of Clinical Genetics, Helsinki University Central Hospital, FI-00029 HUS, Finland

## Abstract

**Background:**

Reduced placental perfusion predisposes to the maternal syndrome pre-eclampsia characterized by systemically reduced perfusion. Considerable data support the role of angiogenic factors in the development of the maternal syndrome. Hypoxia-inducible factor (HIF-1) mediates the cellular responses to hypoxia e.g. by promoting angiogenesis.

**Methods:**

Here we studied whether two single nucleotide sequence variants, c.1744 C>T that changes residue 582 of HIF-1α from proline to serine (P582S) and c.1762 G>A that changes residue 588 of HIF-1α from alanine to threonine (A588T) in the exon 12 of the *HIF1A *gene, are associated with pre-eclampsia. We studied 108 women with pre-eclampsia in their first pregnancy, and 101 controls with normotensive pregnancies. Pre-eclampsia was defined as a blood pressure level of at least 140/90 mmHg in a woman who was normotensive before 20 weeks of gestation, and proteinuria at least of 0.3 g per 24-hour urine collection. The patients and controls were genotyped for variations in the exon 12 of *HIF1A *gene by sequencing

**Results:**

The frequencies of the c.1744 C>T and c.1762G>A sequence variants were not significantly different between women with pre-eclamptic first pregnancies and women with normotensive pregnancies. In addition, two synonymous variants (c.1740G>A and c.1800A>T) were detected at comparable levels in the two groups. All variants were identified in the heterozygous form.

**Conclusion:**

The sequence variants in the exon 12 of the *HIF1A *gene were not associated with pre-eclampsia in the Finnish population.

## Background

Pre-eclampsia, a pregnancy-specific vascular disorder, complicates 3% of pregnancies, and it may threaten the survival of both mother and baby [1]. The onset and clinical course is unpredictable and there are currently no predictive tests or preventive means available in clinical practice. Pre-eclampsia resolves after delivery, which is currently the only existing therapy. It is a heterogeneous disease, commonly mixed presentation of two categories: placental pre-eclampsia with origins primarily in abnormal placental perfusion and maternal pre-eclampsia with origins primarily in pre-existing problems in the mother [2]. Pre-eclampsia shares many common risk factors with atherosclerosis, such as pre-existing hypertension, diabetes, obesity, renal disease and the metabolic syndrome [3]. A large body of evidence also suggests that pre-eclampsia is associated with increased risk of cardiovascular diseases in later life of both mother and baby [4,5]. Twin studies have shown that genetic factors account more than 50% of an individual's susceptibility to pre-eclampsia [6]. Despite intensive research genetic factors predisposing pre-eclampsia are largely unknown [7,8].

Abnormalities in the angiogenic balance have been proposed to as having a major role in the molecular cascade causing maternal endothelial dysfunction and systemically reduced perfusion in pre-eclampsia [9]. Hypoxia-inducible factor (HIF-1) is a transcriptional activator that plays important role in physiologic responses to hypoxia, and the pathophysiology of common human diseases such as ischemic cardiovascular disease, cancer, pre-eclampsia and intrauterine growth restriction (IUGR) [10]. HIF-1 is a heterodimer consisting of an oxygen-regulated HIF1-α subunit and a constitutively expressed HIF1-β subunit [10]. The HIF-1 heterodimer recognizes HIF-response elements within the promoter regions of hypoxia-responsive target genes. More than 100 target genes that are involved in angiogenesis, vascular tone, glucose metabolism, cell proliferation, cell survival and apoptosis are known to be regulated by this mechanism [11]. Under normoxic conditions, the alpha subunit is rapidly degraded by means of ubiquitination and proteosomal degradation [12,13]. HIF1-α protein levels are regulated by the von Hippel -Lindau protein, which targets the N-terminal transactivation domain (N-TAD) within the oxygen-dependent degaradation domain (ODD) of HIF1-α [12,14].

**Figure 1:**
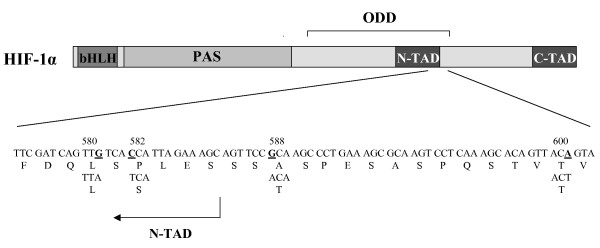
**Structure of the HIF-1α protein, and polymorphisms identified by sequencing the exon 12 of the *HIF1A *gene.** bHLH = basic-helix-loop-helix domain, PAS = Per-Arnt-Sim domain, ODD = oxygen dependent degradation domain, N-TAD = N-terminal transactivation domain, C-TAD = C-terminal transactivation domain. Rs11549465 changes residue 582 of HIF-1α from proline to serine (P582S), and rs11549467 changes residue 588 of HIF-1α from alanine (A) to threonine (T). Variant rs34005929 is a synonymous change of the last nucleotide of codon 580 encoding a leucine (L). Variant changing the last nucleotide of codon 600 (T) is predicted to be a silent polymorphism.

Two non-synonymous single nucleotide sequence variants, rs11549465 and rs11549467, in the exon 12 of the *HIF1A *gene cause amino acid substitution within (rs11549465) or near (rs11549467) the minimal N-terminal transactivation domain (N-TAD) within the ODD that mediates interactions with the von Hippel-Lindau protein, which in turn targets HIF1-α for degradation (Figure 1) [15]. We studied whether these sequence variants, rs11549465 (c.1744 C>T) that changes residue 582 of HIF-1α from proline to serine (Pro582Ser) and rs11549467 (c.1762 G>A) that changes residue 588 of HIF-1α from alanine to threonine (Ala588Thr) in the exon 12 of the *HIF1A *gene, are associated with pre-eclampsia.

## Methods

### Patients

108 women with pre-eclampsia in their first pregnancy, and 101 controls with at least one normotensive pregnancy were included in this study. This is a retrospective analysis of stored DNA samples. The study population has been previously described [16].

Before their first pregnancy, both patients and controls have been healthy and no evidence of renal or autoimmune disease could be detected. Pre-eclampsia was defined using the following criteria; 1) blood pressure (BP) level at least 140/90 mmHg, and 2) proteinuria at least 0.3 g per 24-hour urine collection [17]. The blood pressure was checked by two measurements with at least 6 hours apart and proteinuria was confirmed after gestation week 20. Pre-eclampsia was defined as severe in 95 women who also fulfilled one or more of the following criteria included in the severity predictors of the National High Blood Pressure Education Working Group: systolic BP at least 160 mmHg, diastolic BP at least 110 mmHg, or proteinuria at least 2 g during any 24-hour urine collection [18].

The blood samples were collected between January 1997 and April 1998 from women who, according to discharge records of the Helsinki University Central Hospital, had had severe pre-eclampsia between 1988 and 1998. The control samples were collected from women with uncomplicated pregnancies who gave birth at the Helsinki University Central Hospital. Patients and controls are of Finnish (Caucasian) origin. This study has been approved by the local ethical review committee and an informed consent was obtained from all the study subjects. The clinical characteristics of the patients are presented in Table 1.

**Table 1:** Clinical Characteristics

	**Pre-eclampsia (N = 108)**	**Controls (N = 101)**	
***Variable***	***Mean (95% CI)***	***Mean (95% CI)***	***P***
			
Age (y)	29.5 (28.5, 30.4)	29.9 (28.9.0, 30.9)	NS
BMI (kg/m2)	22.6 (22.0, 23.2)	22.6 (21.6, 23.5)	NS
Systolic blood pressure (mmHg)	171 (168, 175)	119 (117, 121)	<0.0001
Diastolic blood pressure (mmHg)	107 (105, 108)	73 (72, 75)	<0.0001
Proteinuria (g/24 h)	7.5 (5.9, 9.1)		
Birth weight (SD)	-1.5 (-1.7, -1.3)	-0.2 (-0.4, -0.1)	<0.0001
Weeks of gestation	34.5 (33.7, 35.3)	39.6 (39.4, 39.9)	<0.0001

CI = confidence interval

NS = non significant

### Genotyping

Genomic DNA was extracted from 10 ml of peripheral blood using a phenol-chlorophorm method. PCR amplification of the exon 12 of the *HIF1A *gene was performed using forward and reverse primers 5'-CAGAAGCAAAGAACCCAT-3' (F) and 5'-TCAAGAATTTGCGTTAG-3' (R), described by Resar et al. (2005) [19]. The reaction was done in a 50 μl volume containing 70 ng genomic DNA, 30 pmol of each primer, 10 mM Tris-HCl, pH 8.8, 1.5 mM MgCl_2_, 50 mM KCl and 0.1% Triton X-100, 10 nmol of each nucleotide (dNTP) and 0.75 U Dynazyme polymerase-enzyme (Finnzymes Oy, Espoo, Finland). Polymerase chain reaction conditions were as follows: 4 min at 94°C followed by 35 cycles of denaturation step: 30 s at 94°C; annealing step: 30 s at 49°C; elongation step: 30 s at 72°C; and final extension for 10 min at 72°C terminated the reaction after final annealing. Amplifications were performed in a DNA 2720 Thermal Cycler (ABI, Foster City, CA, USA).

Sequencing of the PCR amplicons were performed using Big Dye Terminator kit (version 3.1) supplied by Applied Biosystems (ABI, Foster City, CA, USA). The reactions were run on an ABI 3730 capillary sequencer according to the manufacturer's instructions.

### Statistics and study power

The minor allele frequencies of the r rs11549465 and rs11549467 are 0.092 and 0.018, respectively, in the CEPH population (Utah residents with ancestry from northern and western Europe) . Our sample size has 80% power to detect a relative risk 2.2 for the rs11549465 variant, and a relative risk 2.7 for the rs11549467 variant in the *HIF1A *gene at a significance level α = 0.05. Statistical analyses were performed using GraphPad Prism 4 (GraphPad software Inc., CA, USA). The association between the polymorphisms and pre-eclampsia was estimated by comparing the frequencies in the two study groups. The statistical significance of the different proportions was measured using Fisher's exact test. Parametric and non-parametric tests were used as appropriate for the continuous clinical data. Differences were considered as statistically significant for *P *values <0.05. To determine if genotype frequencies deviate from the the Hardy-Weinberg Equilibrium (HWE) a *Χ*^2 ^test was performed .

## Results

As a result of sequencing exon12 of the *HIF1A *gene and the flanking exon-intron boundaries of *HIF1A *four exonic sequence variants were detected. All the variants in both patients and controls were detected in a heterozygous state. All four SNP genotype frequencies in both patients and controls were in agreement with Hardy-Weinberg equilibrium (p > 0.05.)

No statistical differences were shown when comparing the frequencies of the non-synonymous sequence variants, rs11549465 and rs11549467 between patients and controls. Table 2 shows locations of the detected variants, their effect on the protein, and number of alleles in patients and controls. Numbering of the base positions is relative to the adenine in the ATG startcodon of the *HIF1A *gene.

**Table 2:** Non-synonymous sequence variants within the oxygen-dependent degradation domain of the *HIF1A *gene with pre-eclamptic first pregnancies (N = 108) and women with normotensive pregnancies (N = 101)

**Variation**	**Amino acid**	**No. of alleles in cases**	**No. of alleles in controls**	*P*-value*	**OR (95% CI)**
**rs11549465 (c.1744C>T)**					
**C allele**	Pro582	208	189	0.26	0.56 (0.23–1.38)
**T allele**	Ser582	8	13		
					
**rs11529467 (c.1762G>A)**					
**G allele**	Ala588	212	199	1.00	1.25 (0.28–5.66)
**A allele**	Thr588	4	3		

*Fisher's exact test

In addition to two non-synonymous sequence variants, we found two synonymous sequence variants (Figure 1). Variant rs34005929 (c.1740 G>A), a synonymous change of the last nucleotide of codon 580 encoding a leucine, was detected in two patients but in none of the controls. Variant c.1800 A>T is changing the last nucleotide of codon 600 (Thr), and is predicted to be a silent polymorphism. This variation was detected in four patients and two controls. According to our knowledge this sequence variant has not been reported in public databases.

## Discussion

In this study performed in the genetically homogenous Finnish population, we found that the allele distribution of the two non-synonymous sequence variants, rs11549465 (c.1744 C>T, Pro582Ser) and rs11549467 (c.1762 G>A, Ala588Thr), in the exon 12 of the *HIF1A *gene was not different between women with pre-eclamptic first pregnancies and controls with normotensive pregnancies. We also found two synonymous sequence variants in the exon 12 of *HIF1A *gene: rs34005929 (c.1740 G>A, Leu580Leu) and variant c.1800 A>T (Thr600Thr) which has not previously been reported in public databases.

HIF-1α is known to play role in the normal development and pathology of placenta as a regulator of responses to hypoxia. Accumulation of HIF-1α protein in the pre-eclamptic placentas occurs as a consequence of both increased formation secondary to relative ischemia/hypoxia and reduced degradation after reperfusion/oxygenation due to proteosomal dysfunction [20]. Overexpression of HIF-1α protein in placenta contributes to the dysregulation of numerous genes [21]. Abnormalities in the angiogenic balance have been proposed to as having a major role in the molecular cascade causing maternal endothelial dysfunction and systemically reduced perfusion in pre-eclampsia [9], which makes *HIF1A *an interesting gene in contributing the maternal response to reduced placental perfusion.

Pre-eclampsia originates in the placenta but the target organ is maternal endothelium. Endothelial dysfunction, a central feature in pre-eclampsia, has been suggested to be a part of a more generalized inflammatory reaction [22]. Innate immune response has been linked to the hypoxic response through transcriptional regulation of HIF-1α by transcription factor NF-κB [23].

Pre-eclampsia increases the risk of future ischemic heart disease [24]. This could be an inherent propensity in these women, or vascular damage may have occurred as a consequence of the pre-eclamptic pregnancy. *HIF1A *is one of the genes of importance in the pathways mediating the response to ischemia. The rs11549465 sequence variant was more common in patients who presented with stable exertional angina rather than acute myocardial infarction [25]. These authors suggested that the reduced activity of the variant form of *HIF1A *could reduce plaque neovascularization and the risk of intraplaque hemorrhage and of subsequent acute myocardial infarction [25].

The results of the functional studies of the sequence variants in the *HIF1A *gene in different tissues as well as, the effect of these variants in angiogenesis are inconclusive. The rs11549465 and rs11549467 variants of the *HIF1A *gene have been shown to have enhanced transcription activities in *in vitro studies *under both normoxic and hypoxic conditions [26,27]. Rs11549465 variant was associated with increased tumor microvessel density in head and neck cancer [26], and in prostate cancer [27]. The expression of some down-stream genes that are under the transcription control of HIF-1α (*LDH-5, VEGF, GLUT1*) was not increased in the presence of the rs11549465 variant in non-small cell lung cancer samples studied by immunohistochemistry [28]. In the presence of rs11549465 variant the formation of collaterals in patients with ischemic heart disease was impaired [19].

To the best of our knowledge this is the first study to investigate whether the two non-synonymous sequence variants that lie within or near the N-TAD within the ODD in the exon 12 of the maternal *HIF1A *gene are associated with the pre-eclamptic phenotype.

These sequence variants are interesting because polymorphisms that reduce the activity of HIF-1α as a transcriptional activator could underlie pre-eclampsia by causing inadequate placental vascularisation in the early pregnancy which later leads to hypoxia in the placenta. We are not aware of studies comparing the frequency of these sequence variants between the pre-eclamptic placentas and placentas from normotensive pregancies, or studies measuring the HIF-1α levels in placentas heterozygous of homozygous for these sequence variants.

This retrospective study has several limitations. First, we did not have fetal DNA samples. Consequently, we were neither able to study the effect of fetal/placental genotype nor the maternal fetal genotype interaction. The other limitation is inadequate statistical power to detect small genotypic effects. Under a dominant model, the sample size provides 80% power to detect a genotype relative risk over 2.2, which is much higher than the risk conferred by the majority of susceptibility genes detected to date for complex disorders. For detection of small genotypic effects studies in larger sample sets are warranted. Further studies are also warranted to elucidate effects of the functional polymorphisms in *HIF1A *on the pathogenesis of maternal and placental pre-eclampsia. Pre-eclampsia involves complex interaction between placentation, vascular function and maternal metabolism. Thus, research and recognition of genes, such as *HIF1A*, and thereby biochemical components critical for such interactions increase our understanding of the molecular mechanisms leading from cellular hypoxia to metabolic changes.

## Conclusion

Our data suggest that the sequence variants in the exon 12 of the *HIF1A *gene are not associated with pre-eclampsia in the Finnish population. The study has inadequate statistical power to detect small genotypic effects. Therefore, this gene should be assessed in bigger studies. Further studies are also warranted to elucidate possible fetal genotypic effects.

## Authors' contributions

SA and HL participated in the study design. MK conducted the sequencing. SH, MK and HL analysed the results. SH, MK, and HL wrote the first draft, and all authors the final version of the manuscript. All authors read and approved the final manuscript.

## Pre-publication history

The pre-publication history for this paper can be accessed here:


